# A deep learning method for counting white blood cells in bone marrow images

**DOI:** 10.1186/s12859-021-04003-z

**Published:** 2021-11-08

**Authors:** Da Wang, Maxwell Hwang, Wei-Cheng Jiang, Kefeng Ding, Hsiao Chien Chang, Kao-Shing Hwang

**Affiliations:** 1 Department of Colorectal Surgery, The Second Affiliated Hospital of Zhejiang University School of Medicine, Zhejiang, China; 2 Department of Electrical Engineering, Tunghai University, Taichung, Taiwan China; 3 Department of Electrical Engineering, National Sun Yat-Sen University, Kaohsiung, Taiwan China

**Keywords:** Medical image, Leukemia, Deep learning, Object detection, Classification

## Abstract

**Background:**

Differentiating and counting various types of white blood cells (WBC) in bone marrow smears allows the detection of infection, anemia, and leukemia or analysis of a process of treatment. However, manually locating, identifying, and counting the different classes of WBC is time-consuming and fatiguing. Classification and counting accuracy depends on the capability and experience of operators.

**Results:**

This paper uses a deep learning method to count cells in color bone marrow microscopic images automatically. The proposed method uses a Faster RCNN and a Feature Pyramid Network to construct a system that deals with various illumination levels and accounts for color components' stability. The dataset of The Second Affiliated Hospital of Zhejiang University is used to train and test.

**Conclusions:**

The experiments test the effectiveness of the proposed white blood cell classification system using a total of 609 white blood cell images with a resolution of 2560 × 1920. The highest overall correct recognition rate could reach 98.8% accuracy. The experimental results show that the proposed system is comparable to some state-of-art systems. A user interface allows pathologists to operate the system easily.

## Background

Leukemia is a type of cancer that occurs in the human bone marrow. It causes a large number of abnormal white blood cells to proliferate. Patients with blood cancer can experience anemia, bleeding, purple spots on the skin, fatigue, and an increased risk of infection [1]. The causes of blood cancer are not known, but environmental and genetic factors are important. The density of white blood cells (WBCs) is a measure of the immune system's state and potential risks. In particular, significant variations in the WBC count relative to observed trends could mean that a patient is currently being affected by the antigen due to a malfunctioning immune system. Therefore, WBC counts are quantitative evidence of the progress of the disease.

In a cerebrospinal fluid examination, cerebrospinal fluid is obtained by puncturing the bone marrow and producing a blood smear. A pathologist manually counts each type of cell in each frame under a microscope to check for leukemia and adjust the medication. The differences between each cell are not obvious, so it is difficult to classify cells accurately. This study uses deep learning to detect and count different cells in a blood smear automatically. The proposed system decreases inspection time, and the effect of human factors and the risk of a miscount due to fatigue.

Approach's for existing applications depend on the paradigmatic structure of a multi-stage, cascaded CNNs, as a feature extractor when target objects show large inter-patient variation in shape and size. The feature extractor extracts a region of interest (ROIs) and makes detection on ROIs. The application areas include cardiac, cardiac CT/MRI [2, 3], abdominal object CT segmentation [4], and lung nodule detection [5]. This approach leads to excessive and redundant computational resources on the complicated model; for example, similar features at low-level may be repeatedly extracted by all feature extraction models. A dedicated but effective model is proposed for the simple tasks but with large variations of white blood cells counting in microscopic bone marrow images to tackle this general problem.

By incorporating an attention interface into a generic CNN, model parameters and feature maps are expected to be utilized more efficiently and functionally while reducing the detection model's necessity to solve detection tasks separately globally. The attention interface automatically learns to focus on target objects without additional supervision. The proposed method improves model efficiency yet accuracy comparing to methods based on global training with dense labeling. That is, the proposed method introduces much less significant computational overhead. CNN models with the attention interface can be trained from scratch, similar to fully convolutional network (FCN) models. Similar attention mechanisms have been proposed for natural scene image classification [6] to perform adaptive feature pooling, where predictions are restricted only to a subset of selected image regions.

This study uses a machine learning approach with an attention mechanism explored through the rest of this paper that is a potentially promising advancement over such techniques based on the following reasons: It requires cheaper equipment because captured images are dyed. It provides results almost immediately, unlike conventional image processing methods. The performance of the proposed model is demonstrated in real-time white cell counts in microscopic bone marrow images. The task is challenging due to the low-level feature interpretability of the images, and localizing the object of interest is a critical factor in the successful classification of the cells. We choose to evaluate our implementation on two commonly used state-of-the-arts methods: Faster RCNN [7] and FPN [8]. The results show that the proposed model consistently improves prediction accuracy across different datasets and training sizes while achieving state-of-the-art performance without requiring a global search.

## Methods

**Fig. 1:**
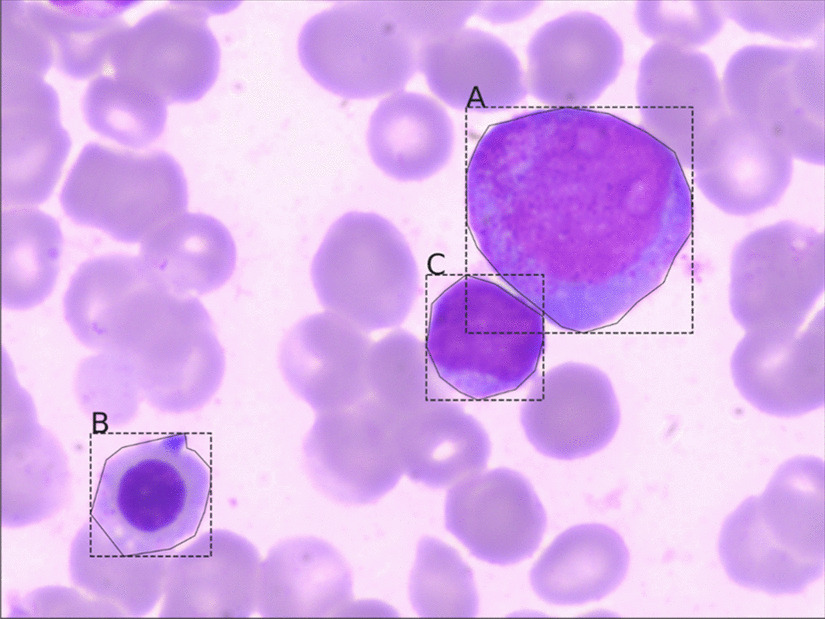
Multiple cells dyed on a blood smear of cerebrospinal fluid

In applications of computer vision, pattern recognition, object localization, and object detection are significant problems. Pattern recognition is used to classify the input image. Object localization identifies the category, position, and size of a single object in the input image. Object detection classifies the location and size of multiple objects. Figure 1 shows different types of cells in a blood smear. Pathologists use dyes to make these cells more distinct for classification. The process identifies the cells' types and frames the cells' locations so that the pathologists can more easily count the numbers in each class in the blood smear.

A variety of deep learning models have been proposed for object detection. These models can be classified into two main categories. One-stage approaches, including YOLO [9] and SSD [10], simultaneously detect the location and classify the target object. Faster RCNN and FPN are two-stage models that first find the region proposal, classify and regress the location to place an anchoring box to frame the target. In general, the former is faster than the latter but less accurate. However, both have a similar structure. The one-stage YOLO and the two-stage Faster RCNN both use an anchor box and bounding box regression, but YOLO uses classification and bounding box regression.

There is a major difficulty in detecting small or adjacent objects because there are only two anchor boxes in a grid, and these predict only one class of object. Faster RCNN detects small objects because a variety of sizes of anchors are used in a single grid. However, real-time detection is not possible using this two-step architecture. Accuracy of recognition is more important than computational efficiency in detecting and counting cells so that two state-deep learning models are used for the proposed system.

### Faster RCNN model

Faster RCNN consists of two parts: a Region Proposal Network (RPN) and Fast R-CNN [11]. These two parts share a hidden layer, which is a deep convolutional neural network. The proposed system uses ResNet-50 [12] as the shared hidden layer. The RPN input is an image, and the output is a set of rectangular region proposals that represent an area that contains an object. After inputting the image, the last layer's feature maps are obtained using the deep convolutional network, and then a sliding window sweeps over the entire feature map. Each point on the feature maps represents an anchor. There are k reference frames in the sliding window. The reference frame is transformed into actual region proposals depending on the parameters' output using the sliding window. During model training, the region proposals' scores are sorted to represent the confidence of the object. The interval of the region proposals' scores in the range of 0.7 to 0.3 is used to train using the Fast R-CNN in a proportion of 1:1. The test identifies the top N region proposals that are output to the Fast R-CNN.

**Fig. 2:**
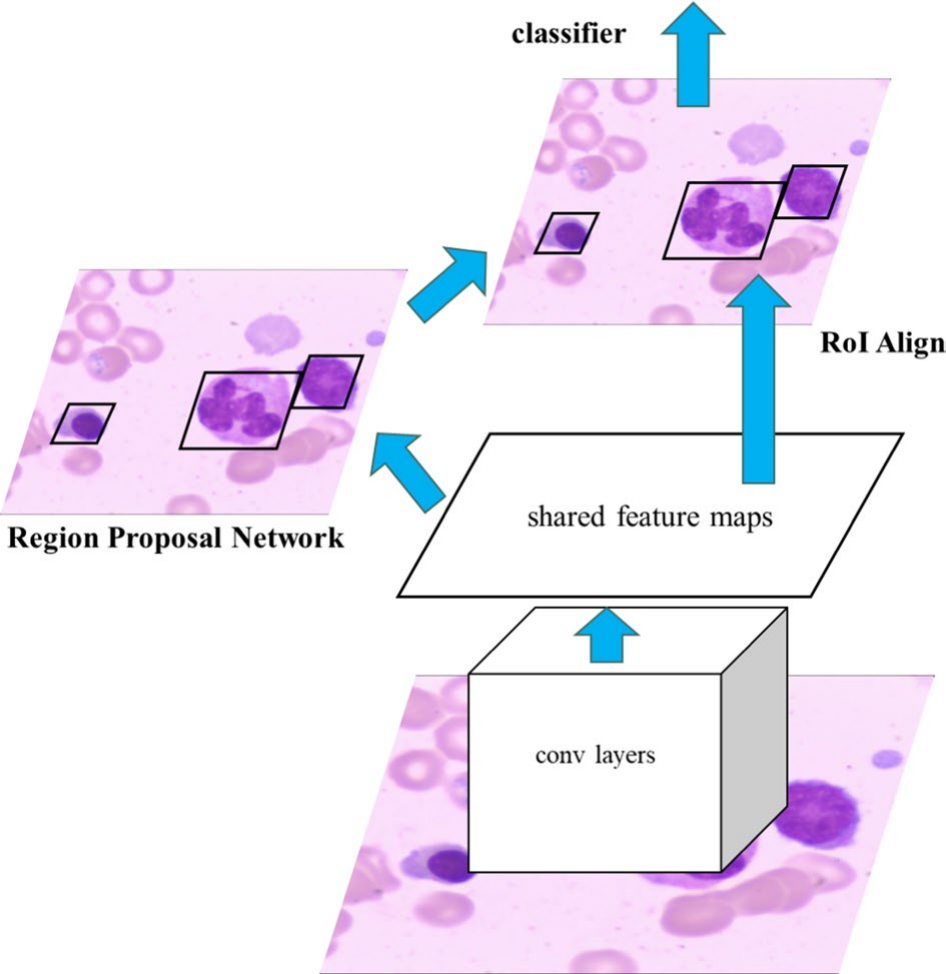
The basic structure of the adjusted Faster R-CNN

The input of the Fast R-CNN is the region proposals extracted using the RPN, and the output is the classification and final location of each region proposal. In the original Fast R-CNN, the region proposals are extracted using RoI Pooling to give RoI's of the same size and then imported into the final network for classification and positional regression. However, RoI Pooling results in the post-extraction features being misaligned with the RoI, so RoI Pooling is replaced with RoIAlign of Mask R-CNN [13]. The architecture of the Faster RCNN is shown in Fig. 2.

### Feature pyramid network model

**Fig. 3:**
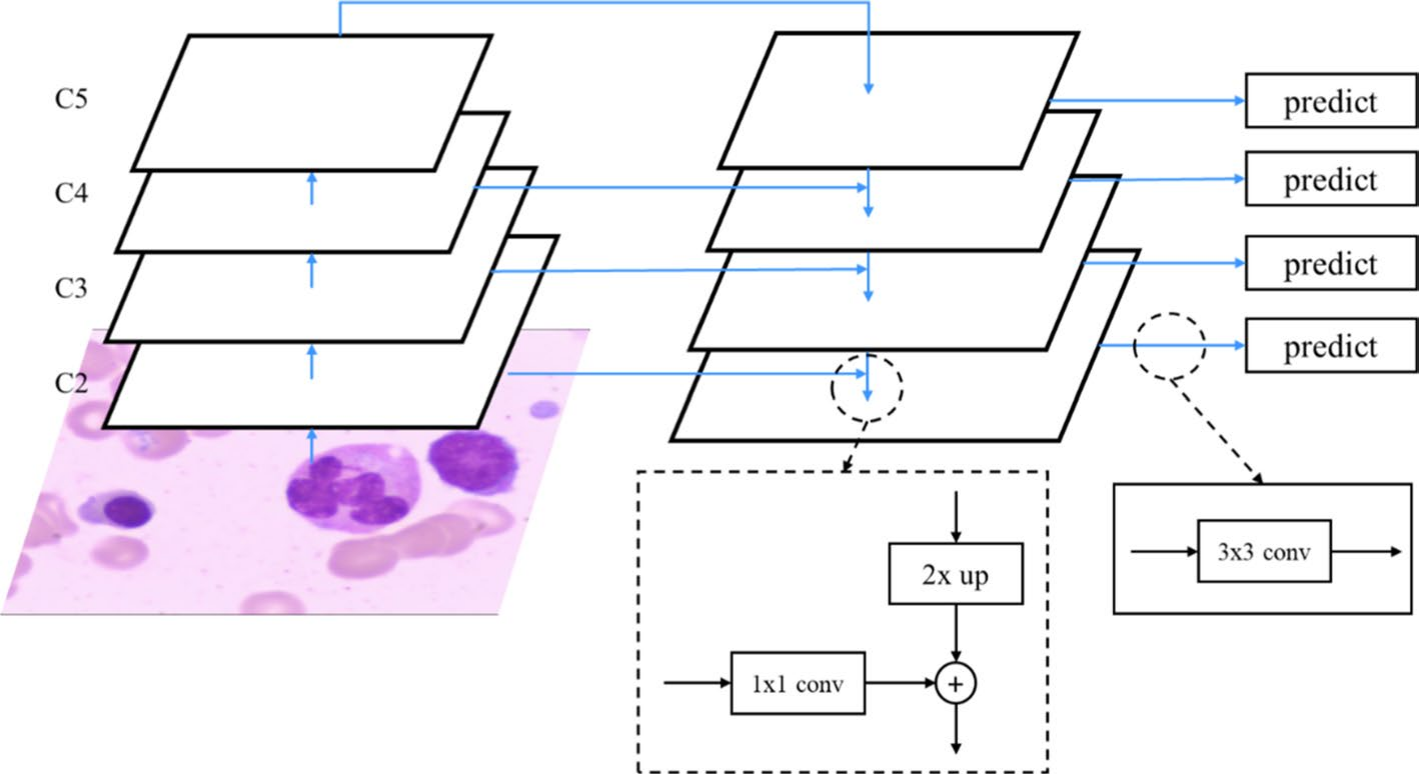
The architecture of a Feature Pyramid Network

A Feature Pyramid Network (FPN) is a deep convolutional neural network. A deep convolutional neural network uses top-level single-scale features for prediction. However, in deep convolutional neural networks, low-level features have less semantic information, but location information is accurate. High-level feature semantic information is plentiful, but information on locations can be eliminated, so some algorithms use multi-scale features for prediction. The input of the FPN is an image, and the output is a multi-scale feature map. The architecture has two parts, as shown in Fig. 3: (1) bottom-up lines and (2) top-down lines and lateral connections.

The bottom-up line is the forward-transferred deep convolutional neural network. Deep convolutional neural networks have many convolutional layers for which the output of feature maps are the same size. These convolutional layers are viewed as the same stage throughout the network, and there can be several steps in the deep convolutional neural network. For a feature pyramid, a pyramid level is defined for each stage. The deep convolutional neural network for the proposed system is ResNet-50. This uses five steps, and the outputs are four feature maps with four different resolutions for layers {C2, C3, C4, C5}, as shown in Fig. 3. High-resolution features in the upper layers are sampled twice from top to bottom, and feature maps of the same size are combined from the bottom up using a 1 × 1 convolution layer. Finally, a 3 × 3 convolution layer is used to eliminate aliasing effects and form a feature pyramid. FPN detects objects similarly to the Faster RCNN. FPN is used for the shared network of RPN and Fast R-CNN. In the RPN part, the output of the FPN is a set of feature maps, so there is a sliding window of RPN on the feature maps of each stage during training. Each sliding window generates the region proposals, and then the Faster RCNN uses the same process of training. In the Fast R-CNN part, the different scales of the pyramid's levels use an RoI of different sizes to extract features for classification and regress the location.

### Attention model

Attention mechanisms are motivated by how humans pay visual attention to different regions of an image. Human visual attention focuses on a specific area with high resolution and perceives the surrounding image as clues in low resolution and then adjusts the focal point or makes an inference. On the other hand, trained attention is enforced by design and categorized as hard- and soft- attention.

In hard attention [14], only a subset of features is selected from a sequence of limited-sight sensing. Therefore, hard attention concentrates on the critical sets and excludes others that are less significant. Hard attention is well suited to these tasks, which rely on very sparse worth-to-be sets over an ample targeting space to mitigate the weaknesses associated with soft attention.

Whereas, hard attention, for instance, iterative region proposal and cropping, is often non-differentiable and relies on reinforcement learning (RL)for parameter updates, which makes model training more difficult. Soft attention is a probabilistic, end-to-end differentiable function.

It utilizes standard back-propagation without the need for posterior sampling. It calculates the distribution of attention using a sequence of sensing over entire images. The resulting probabilities reflect the importance of the resultant attention distribution and produce a weighted encoding feature set. The green dots represent the focus. A soft attention mechanism is fully differentiable and can be easily trained by back-propagation. After the attention process, the softmax function always assigns small values to many insignificant features in the context vector.

In computer vision, attention mechanisms are applied to various problems, including image classification, segmentation, action recognition, and so on. Similarly, non-local self-attention was used to capture long-range dependencies [15]. In medical image analysis, attention models have been exploited for medical report generation [16]. However, although the information to be classified is extremely localized for standard medical image classification, only a handful of works use attention mechanisms [17]. In these methods, either bounding box labels are available to guide the attention, or local context is extracted by a hard-attention model (i.e., region proposal followed by hard- cropping).

### Proposed model for white cell counts

Learning linear transforms for bounding box regression, a reinforcement learning agent with a soft attention mechanism regresses the boxes more broadly. For a specific image, the detection process firstly applies a deep convolutional neural network with an FPN to the entire image to produce a feature map set.

The traditional FPN structure has two parts: a top-down pathway and a lateral connection. However, there is a problem that the feature map is not fully utilized. The location information on the lower level has accuracy, and the higher-level part is rich in feature semantic information. Therefore, a bottom-up structure, a generic CNN, is fully-added to the bottom-up pathway. The top-down pathway and horizontal connections can combine high-level features through upsampling and merge with bottom-up lines through horizontal connections. The bottom-up architecture is the calculation of the feedforward neural network. Each convolutional layer outputs three actions: a prediction operation, an upsampling operation, and a fusion operation with the output of the previous block. After the above operations, the upper and lower layers can be merged, which is compatible with the two's advantages and reduces the output dimension. The overall network structure is shown in Fig. 3. The black dotted frame in the middle is the top-down line and horizontal connection.

The learning of the attention model occurs along the pathway of reinforcement learning. The model takes the feature map as the inputs of keys (K), values (V). the hidden state of the GRU as a query (Q). The Scaled dot product attention for similarity is adopted in the model. The dot product of Q and K divide by a scaling factor $\sqrt {d_{k} }$, where $d_{k}$ is the dimension of Q and K, to prevent the result becoming too accumulatively large as the dimensions of operands are too high.

1 $$\beta \_\alpha = \beta *softmax\left( {\frac{{{\varvec{QK}}^{T} }}{{\sqrt {d_{k} } }}} \right)$$

2 $$\alpha = softmax\left( {\beta \_\alpha \times M} \right)$$

3 $$context\,vector = Attention\left( {{\varvec{Q}},{\varvec{K}},{\varvec{V}}} \right) = \user2{\alpha V}$$

**Fig. 4:**
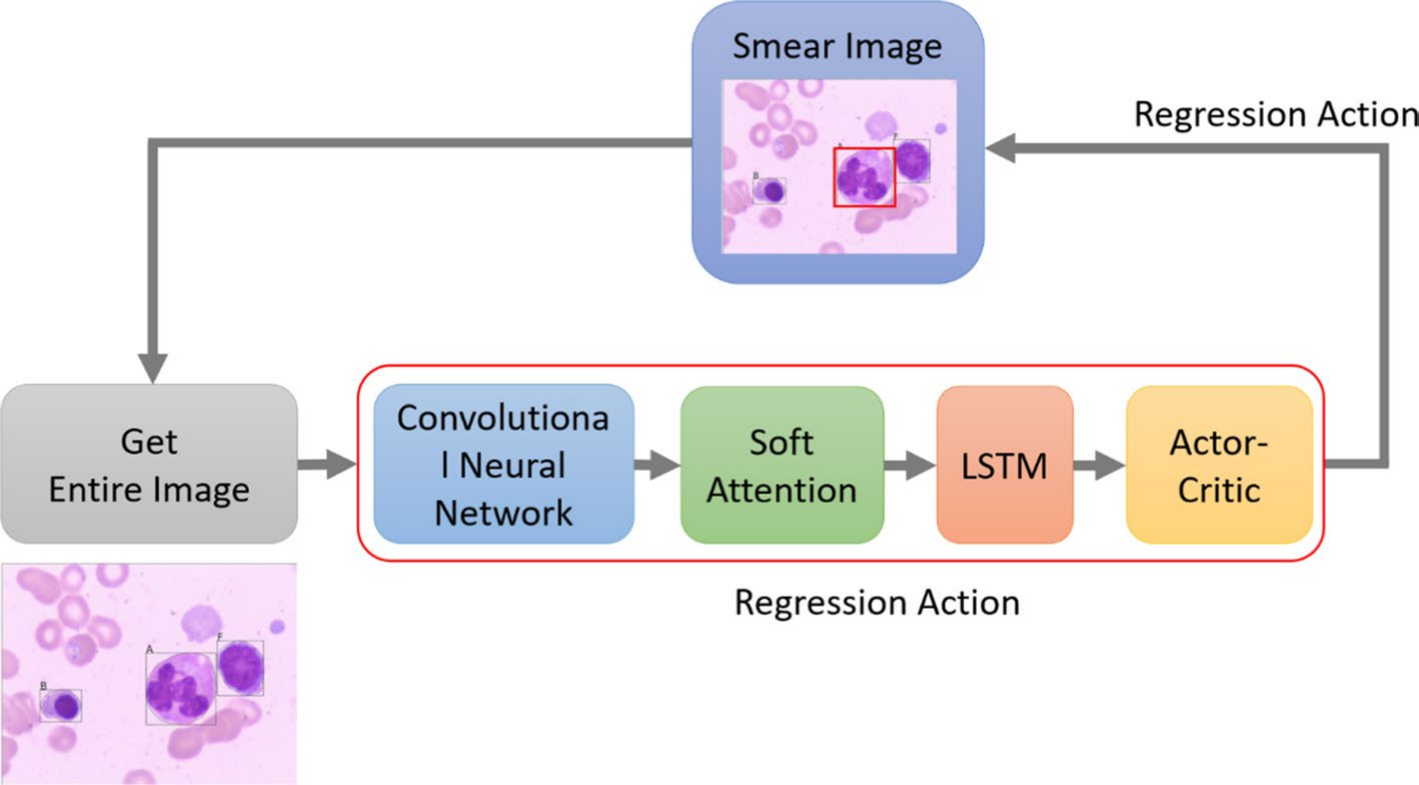
The soft attention mechanism for bounding box regression

All feature vectors for the entire image from the convolution network are assigned attention weighs and used to decide the regression parameters: coordinates, width, and height, as shown in Fig. 4. A Long-Tern-Short-Term Memory (LSTM) is attached to the weighted features stream. A proposal generator also produces a set of proposal bounding boxes on the region pinpointed by the feature with the highest attention score.

Therefore, the process of the proposed local search method is divided into two stages. In the first stage, the local region proposal network (RPN) proposes candidate ROIs from a pinpointing region located in sequence by the attention mechanism. After bounding boxes are generated, the process forks into two branches for classification and positioning regression, respectively. After the RoIs are generated, the local search is conducted by the ranks of IoUs (Interaction of Union) between the gound truths and predictions for classification and bounding box regression.

The classification neural network processes each proposal box separately by extracting the feature maps' features within the box. An actor-critic RL agent executes the classification and bounding box regression. The terminated condition is when the correct classification and the regressing box is close to the ground truth (within a threshold). The result of the classification is used only at the terminal step. Table 2 in the “Appendix” shows the pseudocode for the embedded soft attention mechanism.

## Results

**Fig. 5:**
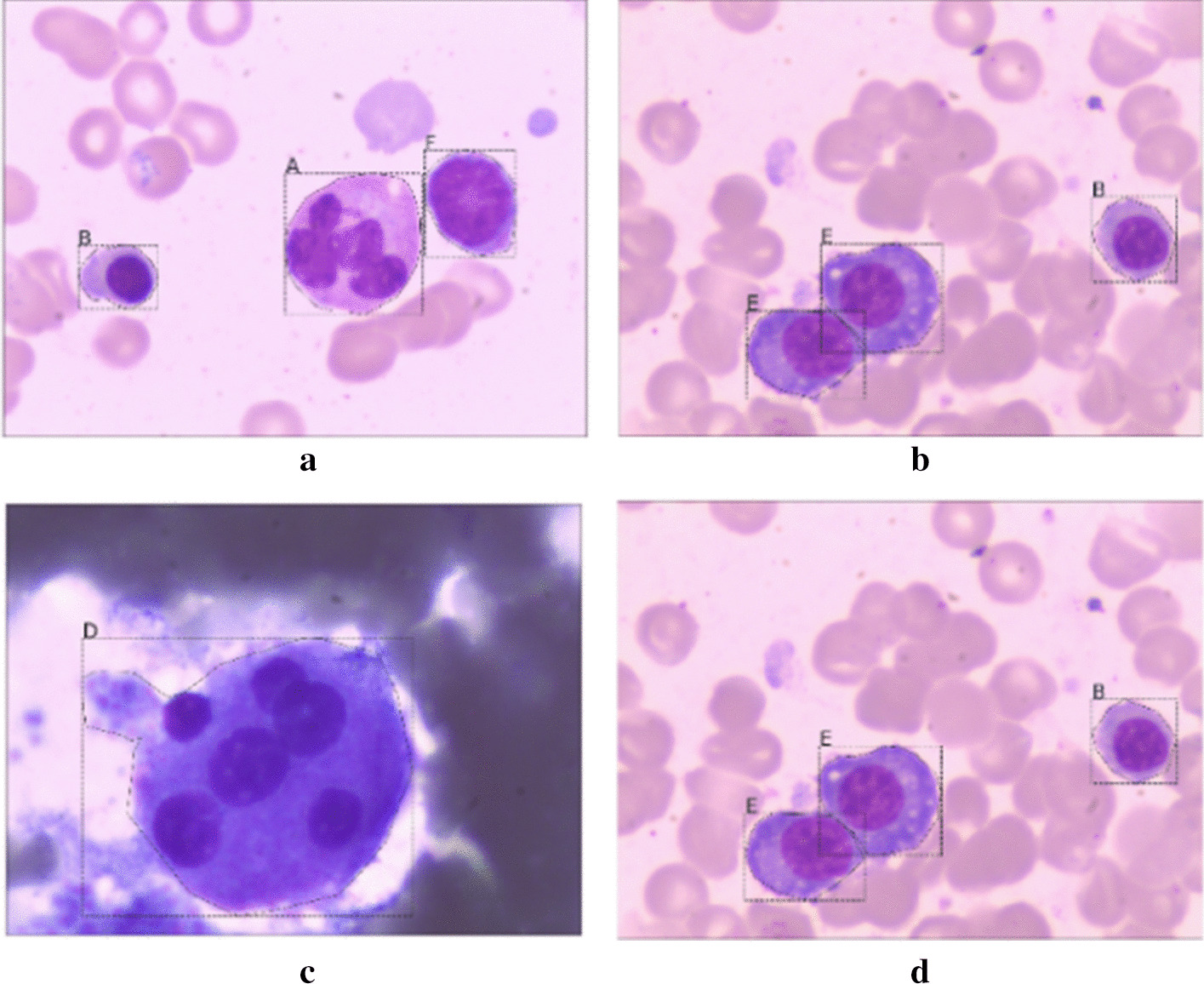
Six types of cells are labeled. "A": Granulocyte; "B": Erythrocyte; "C": Lymphocyte; "D": Megakaryocyte; "E": Plasma cell, "F": Monocyte; for example. (a) Containing "A": Granulocyte; "B": Erythrocyte and "F": Monocyte

Since there is no labeled public dateset available as needed by this work, the data set (MS dataset) is collected from the affiliated hospital of Zhejiang University, China. It is shown in Fig. 5. It contains 609 pictures of 2560 × 1920 pixels, and cells are divided into seven classes: Granulocyte, Erythrocyte, Lymphocyte, Megakaryocyte, Plasma cell, Monocyte, and Others. Each class is shown in Fig. 5a–d. During training, the category of background is added.

In these images, the specific location of the cells is not marked. A VGG Image Annotator [18] is used for digitizing cells' classifications and the annotation for the location. Their formats are then converted to the form of an MS dataset, COCO [19]. The framework is created using PyTorch, which is open source and is provided by Facebook (Francisco et al., 2018), and the hardware GPU, Nvidia GeForce GTX1060. During training for these two models, all images are compressed to 800 × 800 pixels, and the long edge is compressed by the reduction ratio for the short edge. All data is divided into 90% training data and 10% test data.

**Fig. 6:**
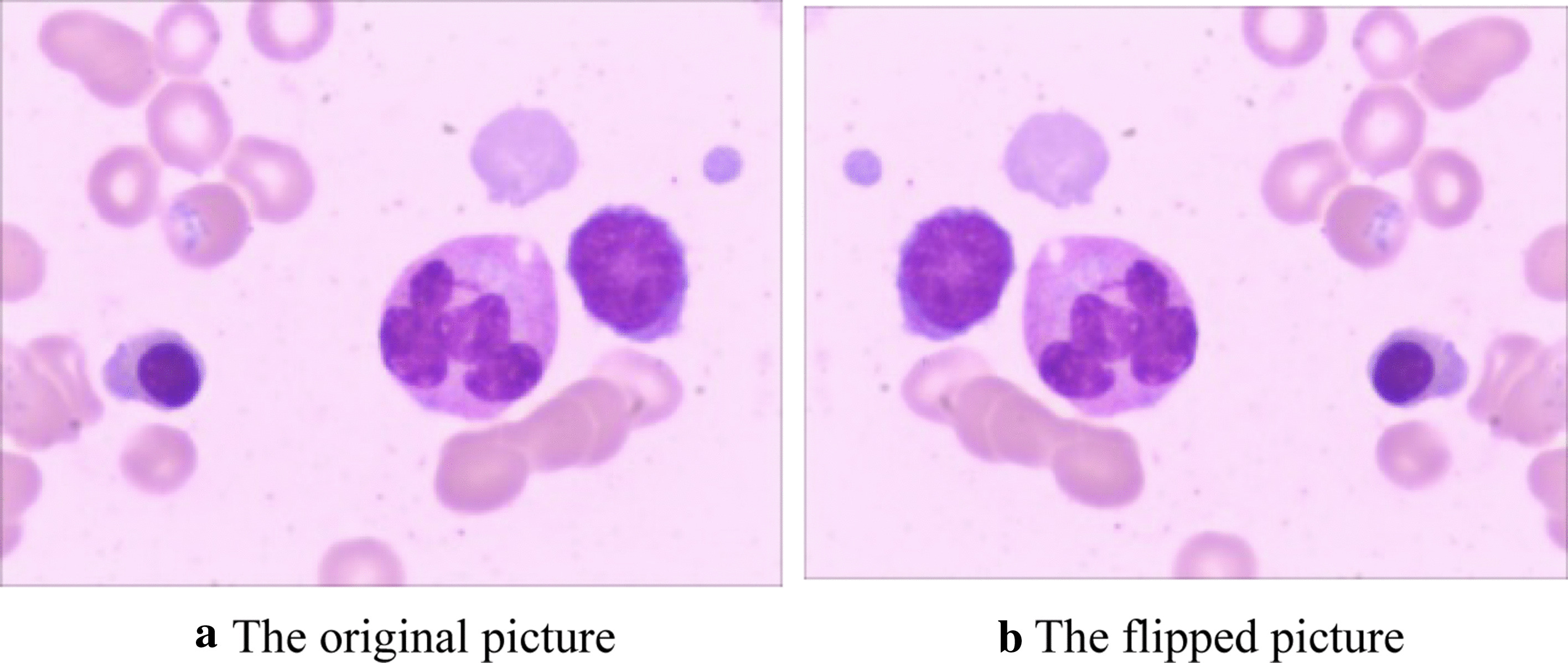
An example of data augmentation using an original image

Cells do not have a specific orientation, so operations, such as reflection, rotation, and shearing the cells' images, are used to increase the training set's size. The training data set's size is increased from 609 to 1218 images by a reflection (flip) operation, as shown in Fig. 6. Augmentation does not increase the number of samples for each blood cell class so that the dataset remains balanced.

The metrics used to characterize an object detector's performance for the MS dataset are Average Precision (AP) and Average Recall (AR). AP is averaged overall categories. This is known as "mean average precision" (mAP). AR is the maximum recall for a fixed number of detections per image, averaged over categories and IoUs. AR is related to the same name metric, which is used in proposal evaluation but is computed on a per-category basis.

**Fig. 7:**
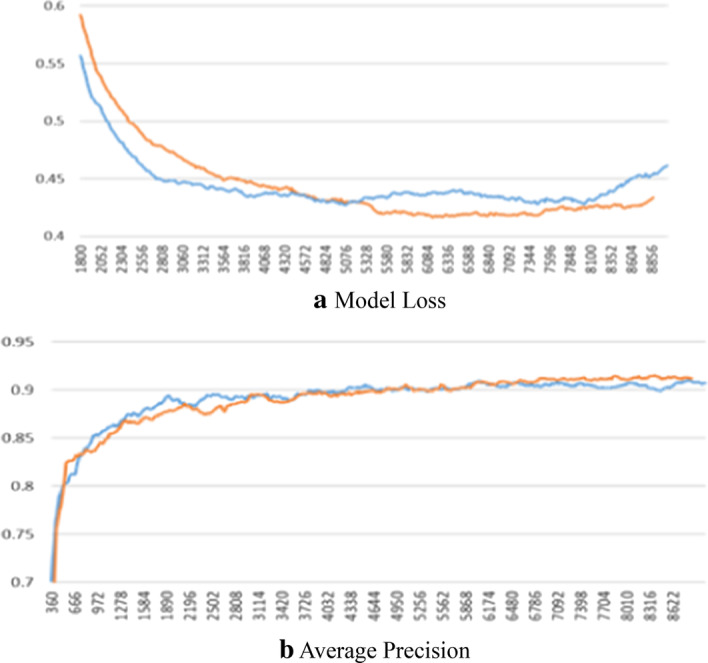
A comparisons of the loss and average precision for the improved model and Faster-RCNN

The model uses end-to-end training with back-propagation and stochastic gradient descent. Each mini-batch includes two images and 30,000 training iterations. Table 1 shows the comparison results between the improved model, the Faster-RCNN, and the FPN substituting to the Faster-RCNN (called the FPN method in the following) as the feature extractor. The comparisons used the evaluation indicators for the MS dataset. FPN uses pyramids to improve performance and performs better than the Faster RCNN using the MS dataset. The improved model performs better than FPN in this study, explaining why cells' sizes are not variable as the MS dataset targets. The experimental results demonstrate that the Faster RCNN performs better than FPN in terms of Average Precision (AP) and Average Recall (AR), but more training time and testing time are required for FPN. Figure 7 shows the comparisons of the loss and average precision for the improved model and its original.

**Table 1:** The performance of two models are compared using the metrics, MS COCO's Average Precision (AP) and PASCAL's APx0 (IoU over a threshold of 0.x), and AP (APS, APM, APL) for different object sizes (Small,, Medium, Large)

Models	AP	AP50	AP75	APS	APM	APL
Improved model	0.744	0.853	0.863	0.988	0.831	0.755
Faster RCNN	0.715	0.844	0.838	0.988	0.830	0.725
FPN	0.678	0.800	0.795	0.950	0.826	0.689

**Fig. 8:**
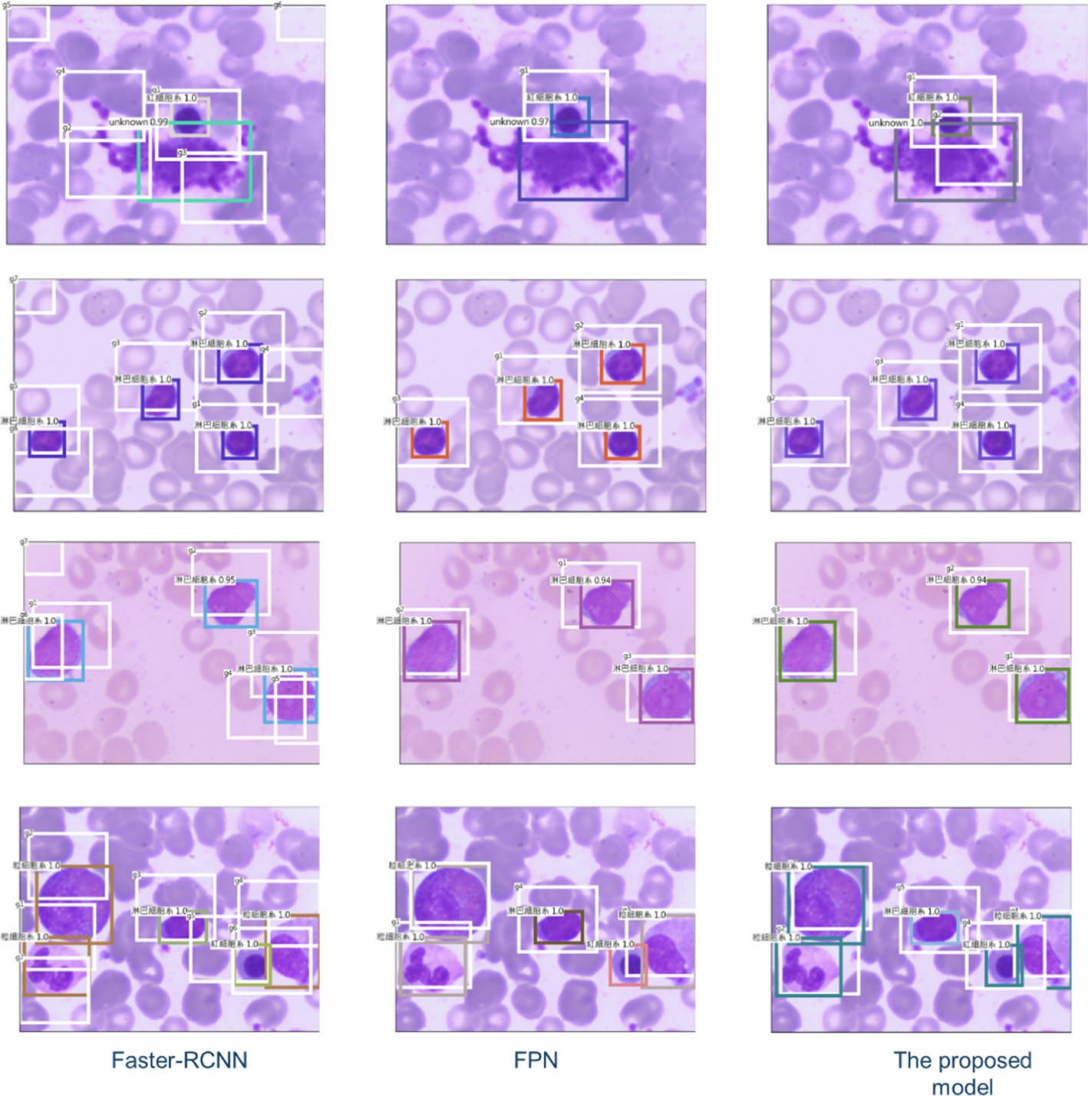
The visualized results in the experiments; the first column is from Faster-RCNN, the second is the one of substituting FPN into Vgg of the Faster-RCNN; the third one is the result from the proposed model

**Fig. 9:**
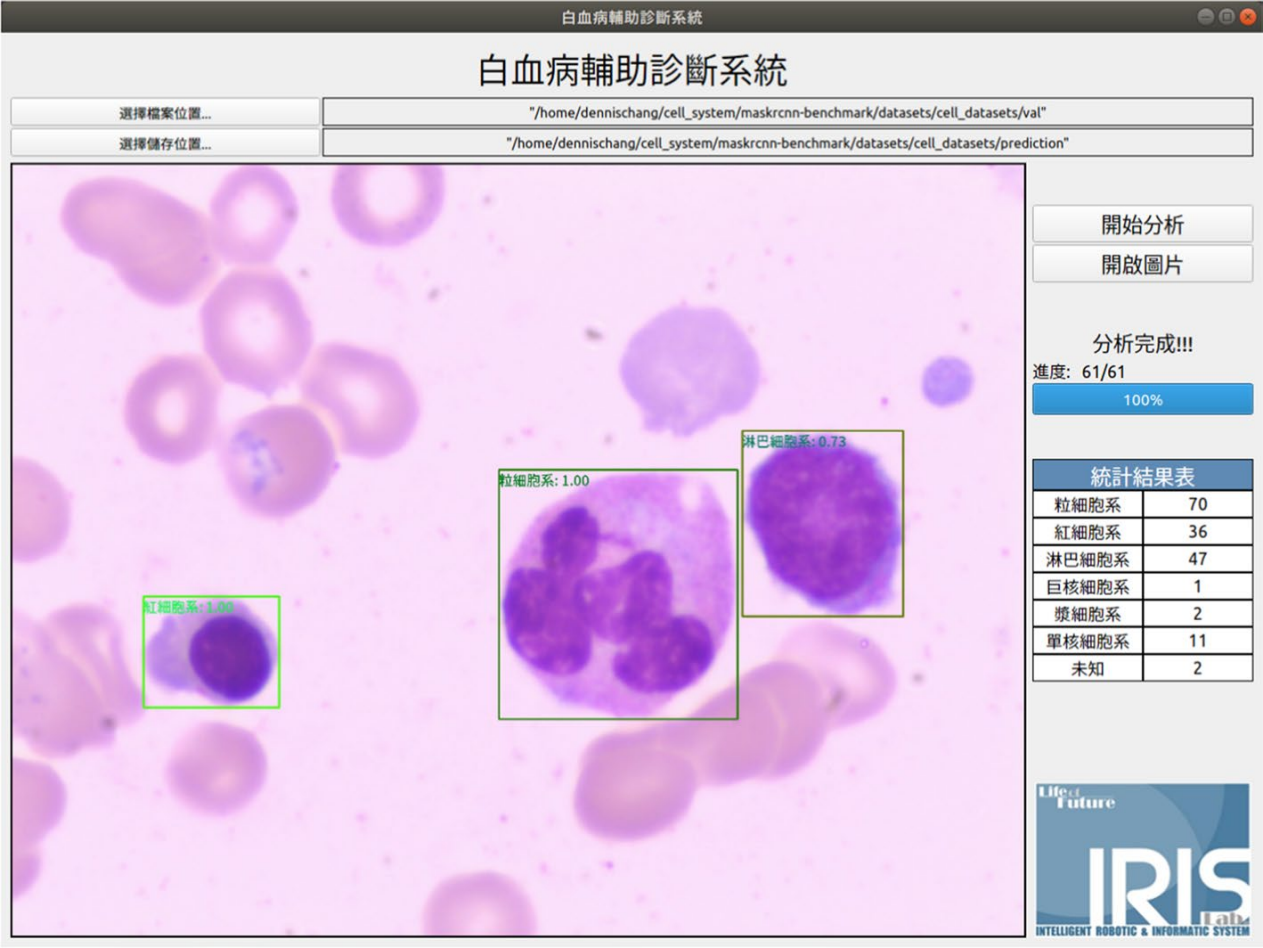
The interface for the proposed system

The values for AP and AR are higher for Faster RCNN, so the models' computational efficiency is verified. Faster RCNN is used as the core algorithm for an auxiliary diagnosis system for leukemia. Practically, the confidence threshold for the detection frame is set to a greater than 0.7 of the output, and multi-class non-maximum suppression is used to allow more reliable final detection. After analysis, the statistical results for each type of cell are output, and the analyzed images are available for comparison. Figure 8 shows the visualized results in the experiments. To allow user-friendly operation, PyQt5 [20] is used to construct a convenient user interface for the pathologists who are not familiar with the deep learning model shown in Fig. 9.

## Discussion and conclusions

This study uses a deep learning model to detect and count white blood cells. The experimental results show that the proposed system is comparable to state-of-art systems. The proposed model uses an improved Faster-RCNN model to classify the white blood cells in the dataset more accurately at 74.4%, 85.3%, 86.3%, 98.8%, 83.1%, and 75.5% different IoU levels with image-level data. The proposed method is suitable for datasets of WBCs and a wide variety of other cells and tumor cells. The method allows faster iteration cycles, lower labor costs, and better patient outcomes and allows machine learning to be meaningfully applied in healthcare.

There remained problems with cell detection, such as multiple classes, more varied lighting conditions, and new cell types. The following limitations apply to this study. A dataset with more varied cell images of interest is required to produce more confident predictions and counts. Most of the images are acquired under the same lighting and microscopy conditions. Images that involve more varied ambient conditions are required to verify the generalizability of the proposed model.

## Data Availability

The limited data set (MS dataset) is collected from the affiliated hospital of Zhejiang University, China, and is not admitted to public access for the reason of confidentiality.

## Appendix

See Table 2.

**Table 2:** Pseudocode of the embedded Soft Attention Mechanism; *t*_*max*_ the maximum number of the training episodes of each regression, *T*_*max*_ is the number of training termination

Pseudocode of the embedded Soft Attention Mechanism
Initialize
Global variables, model parameters $\theta ,\theta_{v}$ and counter *T* = 0
Parameters of RL agents: $\theta^{{\prime }} ,\theta_{v}^{{\prime }}$, *t* ← 1
Repeat
Initialize the gradients $d\theta \leftarrow 0,d\theta_{v} \leftarrow 0$
Synchronize the RL agents $\theta^{{\prime }} = \theta ,\theta_{v}^{{\prime }} = \theta_{v}$
Initialize the initial states of LSTM $c_{0} ,h_{0}$
$$t_{start} = t$$
Take the entire image $x_{t}$
Repeat
FPN extracting the feature vectors $v_{t}$ of $x_{t}$
Using $v_{t}$, the previous state of LSTM $h_{t - 1}$
to obtain an attentive state $Z_{t}$
Input $Z_{t} ,c_{t - 1} ,h_{t - 1}$ to LSTM
LSTM output $$h_{t}$$
$$s_{t} \leftarrow h_{t}$$
Take regression action $a_{t}$ by $\pi (a_{t} |s_{t} ;\theta^{{\prime }} )$
Obtain reward $R_{t}$ and a new image $x_{t + 1}$
$$t \leftarrow t + 1$$
$$T \leftarrow T + 1$$
Until *r*eaching the terminal state $s_{t}$ *or* $t - t_{start} = t_{max}$
$$G = \left\{ {\begin{array}{*{20}l} 0 \hfill & {terminal\,states_{t} } \hfill \\ {V\left( {s_{t} ;\theta_{v}^{{\prime }} } \right),} \hfill & {non - terminal\,states_{t} } \hfill \\ \end{array} } \right.$$
$${\mathbf{for}}\,\, i \in \left\{ {t - 1, \ldots ,t_{start} } \right\}{\mathbf{do}}$$
$$G \leftarrow R_{t} + \gamma G$$
Calculate the gradients $\theta_{v}^{{\prime }}$: $d\theta_{v} \leftarrow d\theta_{v} + \frac{{\partial \left( {G_{t} - v\left( {s_{t} ;\theta_{v}^{{\prime }} } \right)} \right)^{2} }}{{\partial \theta_{v}^{{\prime }} }}$
$$\theta^{{\prime }}$$: $$d\theta \leftarrow d\theta + \nabla_{{\theta^{\prime}}} \log \pi (a_{t} |s_{t} ;\theta^{{\prime }} )\left( {G_{t} {-} V\left( {s_{t} ;\theta_{v}^{{\prime }} } \right)} \right) + \beta \nabla_{{\theta^{{\prime }} }} H\left( {\pi \left( {s_{t} ;\theta^{{\prime }} } \right)} \right)$$
End for Until $T > T_{max}$

## Abbreviations

WBC: White Blood Cells
ROIs: Region of Interest
FCN: Fully Convolutional Network
YOLO: You Only Look Once
FPN: Feature Pyramid Network
RL: Reinforcement Learning
K: Keys
V: Values
Q: Query
LSTM: Long-Tern-Short-Term Memory
RPN: Region Proposal Network
IoU: Interaction of Union
AP: Average Precision
AR: Average Recall
